# Clinical Manifestations of Hyperandrogenism and Ovulatory Dysfunction Are Not Associated with His1058 C/T SNP (rs1799817) Polymorphism of Insulin Receptor Gene Tyrosine Kinase Domain in Kashmiri Women with PCOS

**DOI:** 10.1155/2021/7522487

**Published:** 2021-12-06

**Authors:** Shayaq Ul Abeer Rasool, Sairish Ashraf, Mudasar Nabi, Shariq R. Masoodi, Khalid M. Fazili, Shajrul Amin

**Affiliations:** ^1^Department of Biotechnology, University of Kashmir, Srinagar, India; ^2^Department of Biochemistry, University of Kashmir, Srinagar, India; ^3^Department of Endocrinology, Sher-i-Kashmir Institute of Medical Sciences, Srinagar, India

## Abstract

**Background:**

Polycystic ovary syndrome (PCOS) is the most common endocrine metabolic disorder affecting premenopausal women. Besides primary features like anovulation, hyperandrogenism, and polycystic ovaries, women with PCOS present with multiple metabolic, cardiovascular, and psychological disorders. The etiology is multifactorial and the different genetic variants are suggested to play an important role in pathogenesis. Insulin resistance is a ubiquitous finding in PCOS and SNPs in genes involved in the insulin signaling pathway are possible candidates that can explain the development of clinical manifestations of PCOS.

**Aim:**

We aimed to investigate the association of INSR His1058 C/T (rs1799817) single nucleotide polymorphism with PCOS in Kashmiri women. The genotypic-phenotypic correlation of the tested SNP with hyperandrogenism, ovulatory dysfunction, and metabolic markers was evaluated.

**Results:**

The allele frequency (OR = 1.00, 95% CI = 0.67–1.48, *χ*^2^ = 0.01, *P*=0.99) and genotype distribution (*χ*^2^ = 3.73, *P*=0.15) in INSR C/T polymorphism were comparable with controls. No significant association was found with PCOS in dominant (*P*=0.194), recessive (*P*=0.442), and homo vs. het. (*P*=0.5) genotype models. Genotype-phenotype correlation analysis revealed that variant TT genotype had significantly higher HOMA (*P*=0.029) and reduced insulin sensitivity QUICKI (*P*=0.037) values. There was no significant variation in the prevalence of hirsutism, acne, alopecia, menstrual disturbances, acanthosis nigricans, and obesity (all *P* > 0.05) in different INSR C/T genotypes.

**Conclusion:**

The INSR C/T SNP (rs1799817) does not increase the risk of PCOS in Kashmiri women. This SNP is unlikely to play a significant role in the development and manifestation of clinical symptoms of polycystic ovary syndrome.

## 1. Introduction

PCOS, a multidimensional disorder, affects reproductive, endocrine, metabolic, cardiovascular, and psychological health of affected women [1]. It is manifested as a spectrum of symptoms including oligomenorrhea, hirsutism, infertility, acne, and obesity [2]. The etiology is considered polygenic and multifactorial, and family-based studies suggest a strong genetic component [3, 4]. The genetic variants have been extensively investigated to understand their contribution in predisposing women to PCOS [5, 6]. The insulin resistance and hyperinsulinemia, commonly found in 40–80% PCOS women, are suggested to play important role in metabolic and reproductive disturbances in women with PCOS [7]. The subnormal response to insulin indicates defects in insulin receptor or postreceptor signaling may be involved in pathogenesis of PCOS. In PCOS, no change in the expression or affinity of INSR for insulin has been observed but the phosphorylation status and downstream signaling are affected [8, 9]. This indicates defects in INSR, especially tyrosine kinase domain in *β* subunit. Insulin receptor gene (INSR) consists of 22 exons and is located on the short arm of chromosome 19. INSR, a heterotetramer, has two extracellular *α* subunits and two transmembrane *β* subunits. The *α* subunit, encoded by exons 1–11, has insulin binding domain and *β* subunit, encoded by exons 12–22, has tyrosine kinase domains [10]. The genetic variants in exons 17–21, codes for tyrosine kinase domain, have been investigated to understand downstream signaling alterations postinsulin binding [11, 12]. A C/T single nucleotide polymorphism (rs1799817) at ATP binding site in tyrosine kinase domain has been particularly investigated in many disorders [13]. This SNP has been investigated for association with type 2 diabetes, hypertension, colorectal cancer, nonalcoholic fatty liver disease, and PCOS [14–17]. The genome wide association study by Shi et al. [18] identified region near INSR gene as possible PCOS susceptibility locus. So, this gene becomes important target of investigation to understand the pathogenesis of PCOS. Some candidate gene association studies reported association of INSR C/T SNP with PCOS in Caucasian and Chinese women but other studies in Asian and Caucasian ethnicities have failed to replicate this association [13, 19–21]. In Indian women, Mukherjee et al. showed strong association of this variant with hyperandrogenemia in lean PCOS women but not in obese PCOS women [15]. Thangavelu et al. reported no association of this SNP with PCOS in South Indian women [22].

In this study, we aimed to investigate the association of INSR C/T SNP (rs1799817) with PCOS in North Indian Kashmiri women. We also investigated phenotypic-genotypic association of this genetic variant and its effect on endocrine and metabolic manifestations was studied in PCOS.

## 2. Materials and Methods

### 2.1. Recruitment of Subjects

The women visiting endocrinology outpatient clinic of Sher-i-Kashmir Institute of Medical Sciences (SKIMS), India, for PCOS-related symptoms from June 2015 to October 2018 were evaluated for PCOS. This case-control study involved 349 ethnic Kashmir women from 16–30 ages. 249 women diagnosed with PCOS consented to participate in the study and were recruited for the study. The diagnosis of PCOS was done according to the revised 2003 Rotterdam European Society for Human Reproduction and Embryology and American Society for Reproductive Medicine (ESHRE/ASRM) criteria [23]. The women were diagnosed with PCOS if at least two of the following three features were present: (1) oligo- and/or anovulation defined by menstrual cycles of <21 days, >35 days, or less than 6–9 menstrual cycles in a year and amenorrhea as absence of at least 6 menstrual cycles, (2) clinical and/or biochemical signs of hyperandrogenism (Ferriman Gallwey score ≥8 or total testosterone ≥50 ng/dL, and (3) polycystic ovaries ≥12 antral follicles measuring 2–9 mm in diameter or ovarian volume >10 mL in one or both ovaries by transabdominal ultrasonic examination [24]. All participants were screened for PCOS mimicking disorders like hyperprolactinemia, thyroid dysfunction, Cushing's syndrome, congenital adrenal hyperplasia, and androgen-secreting ovarian/adrenal tumors. The control group consisted of 100 age-matched healthy volunteers recruited from various faculties of University of Kashmir, Srinagar, India. The eligibility criteria for the control group included age-matched healthy volunteers with regular menstrual cycles, no clinical or biochemical hyperandrogenism, and no acne on physical examination. They also had no history of endocrine or autoimmune disorders and have not undergone surgery to the pelvic region [5]. All subjects were ethnic Kashmiris/North Indians living in Kashmir province of India.

### 2.2. Ethics Statement

This study was approved by the institutional ethics committee under ethical approval no. SIMS 1–31/IEC-SKIMS/2013/6592. Subjects were recruited after written informed consent was obtained from them.

### 2.3. Anthropometric and Clinical Evaluation

The clinical and family history including menstrual history, acne, alopecia, and acanthosis nigricans was taken from the cases and controls. The anthropometric parameters like height, weight, waist circumference, and hip circumference were recorded. The anthropometric variables were used to calculate body mass index (BMI: weight (kg)/height (m^2^)), waist to hip ratio (WHR: waist circumference [cm]/hip circumference [cm]), and waist to height ratio (WHtR: waist circumference (cm)/height (cm)). The clinical parameters like systolic and diastolic blood pressure (SBP and DBP), hirsutism, acne, alopecia, and acanthosis were examined. Height was measured in standing position without shoes using a height measuring scale. Weight was measured by digital weighing scale (Krupps, India) with light clothing and without shoes. Waist circumference was measured in standing position as the minimum value between the iliac crest and the lateral costal margin at the end of a gentle expiration, and hip circumference was calculated as the maximum value over the buttocks. Blood pressure (BP) was measured in a relaxed sitting position after 5–10 min rest by Diamond Mercurial sphygmomanometer blood pressure monitor. Ferriman–Gallwey scoring system was used to evaluate the hirsutism in participants. In this nine androgen sensitive body parts examined and scored from 0 (no hair) to 4 (male pattern hairiness), a cumulative score of ≥8 was taken as cutoff for hirsutism.

### 2.4. Biochemical and Hormonal Assessment

After a 12 hr overnight fast, blood samples were obtained from the participants in the early follicular phase (Days 2-3) of the spontaneous menstrual cycle or withdrawal bleeding with progesterone for subjects with amenorrhea. The blood vials were placed on ice immediately, and serum was separated by centrifugation at 3000 rpm for 10 min at 4°C within 2 h. Serum aliquots were stored at −80°C for further analysis. The serum concentration of luteinizing hormone (LH), follicle-stimulating hormone (FSH), total testosterone (TT), thyroid-stimulating hormone (TSH), and prolactin (PRL) was measured by Radioimmunoassay (RIA) on Beckman Coulter UniCelDxl 800 (Access Immunoassay System) using RIA kits (Immunotech s.r.o, Prague, Czech Republic). Enzyme-linked immunosorbent assays were used to measure sex hormone-binding globulin (SHBG), androstenedione, dehydroepiandrosterone sulfate (DHEAS), and fasting insulin using ELISA kits (Calbiotech, CA, USA, and DGR Instruments GmbH, Marburg, Germany).

The free androgen index (FAI) was derived using the following formula:

(1) $$\begin{array}{c} \text{FAI}=\frac{\text{total testosterone nmol}/\text{L}}{\text{SHBG nmol}/\text{L}}\times \;100. \end{array}$$

The glucose and lipid indices were measured in study subjects. The glucose and insulin were measured in fasting state and 2nd hour glucose after 75 g oral glucose tolerance test (OGTT). The levels of glucose were measured by the glucose dehydrogenase method. Insulin resistance was estimated by the homeostatic model assessment of insulin resistance (HOMA-IR) method derived using the following formula:

(2) $$\begin{array}{c} \text{HOMA}=\frac{\text{fasting glucose }\left(\text{mg}/\text{dL}\right)\times \text{fasting insulin}\left(\mu \text{IU}/\text{mL}\right)}{405}. \end{array}$$

The insulin sensitivity was estimated by the quantitative insulin sensitivity check index (QUICKI) according to the following formula:

(3) $$\begin{array}{c} \text{QUICKI}=\frac{1}{\log\text{ fasting insulin}\left(\mu \text{IU}/\text{mL}\right)+\log\text{ fasting glucose}\left(\text{mg}/\text{dL}\right)}. \end{array}$$

The lipid accumulation product (LAP) was calculated using the following formula:

(4) $$\begin{array}{c} \text{LAP}=\left(\text{waist circumfrence}-58\right)\times \text{triglycerides}. \end{array}$$

The body adipose index (BAI) was calculated using the following formula:

(5) $$\begin{array}{c} \text{BAI}=\frac{\text{hip circumfrence}\left(\text{cm}\right)}{{\text{height}}^{1.5}}-18. \end{array}$$

The biochemical parameters like fasting serum lipid profile (cholesterol (CHOL) and triglycerides (TG), urea, uric acid, creatinine, alanine aminotransferase (ALT), and aspartate aminotransferase (AST) were determined by enzymatic methods using Erba bioassay diagnostic kits and analyzed on Erba Chem7 biochemistry analyzer (ERBA Diagnostics, Manheim, Germany) [5].

### 2.5. Genotyping

The genomic DNA was isolated from peripheral blood leukocytes by using QIAamp DNA mini kit (QIAGEN, Hilden, Germany). The DNA concentration was measured at OD260 and purity was checked by the OD260/OD280 ratio using nanodrop 2000c spectrophotometer (Thermo Fisher Scientific, Wilmington, USA) and integrity was checked by subjecting DNA to electrophoresis on 1% agarose gel. For genotyping, INSR C/T polymorphism was analyzed by polymerase chain reaction restriction fragment length polymorphism (PCR-RFLP). The PCR amplification was carried out according to Siegel et al. [21]. The forward 5′- CCAAGGATGCTGTGTAGATAAG -3′ and reverse 5′- TCAGGAAAGCCAGCCCATGTC -3′ primer were used for amplification. PCR reaction conditions consisted of an initial denaturation at 94°C for 5 min followed by 35 cycles, and each cycle consisted of denaturation at 94°C for 30 s, annealing at 48.8°C for 40 s, and extension at 72°C for 40 s, and final extension at 72°C for 7 min using Agilent Surecycler 8800 (Agilent, Santa Clara, USA). The PCR product was subjected to restriction fragment length polymorphism (RFLP) and digested by 10 U of PmlI restriction enzyme (New England Biolabs, Wilmington, USA) at 37°C. The digested products were separated on 2.5% agarose gel and visualized on Odyssey FC imaging system (LI-COR Biosciences, USA). The C allele resulted in an undigested PCR product of 317 bp, and T allele resulted in a digested PCR product with two fragments of 274 bp and 43 bp.

### 2.6. Statistical Analysis

All continuous variables were presented as mean ± standard deviation and categorical variables as numbers and percentages. Clinical, anthropometric, hormonal, and metabolic variables were compared between PCOS and controls and genotype groups by unpaired student *t*-test and nonparametric variables were compared by chi-square test. Pearson chi-square (*χ*^2^) test was used to reveal differences in allele and genotype frequencies and test deviations of genotype distribution from Hardy–Weinberg equilibrium between PCOS and controls. Odds ratio and 95% confidence intervals were calculated to test relative risk of dominant, recessive, and additive models. One-way analysis of variance (ANOVA) independent standard weighted-means analysis was used to compare multiple groups in additive genotype model followed by post hoc Tukey HSD test for intergroup association. The statistical analysis was performed using statistical computation software VassarStats (http://vassarstats.net/). The bar graph was generated by Sigma plot 10.0 software. *P* value of <0.05 was considered as statistically significant.

## 3. Results

We found C allele in 77.51% and 77.5% and T allele in 22.48% and 22.5% cases and controls, respectively. The allele frequency of PCOS group was not statistically significant (OR = 0.99, CI = 0.67–1.48, *χ*^2^ = 0.01, *P*=0.99). The CC genotype was present in 62.65% PCOS cases and 67% controls. The 29.71% PCOS cases and 21% controls had heterozygous CT genotype while TT genotype was found in 7.6% PCOS cases as compared to 12% controls. No significant association was found between PCOS and controls in genotype frequencies (*χ*^2^ = 3.73, *P*=0.15). The genotypes were compared in different genotype association models like dominant (CC + CT vs. TT), recessive (CC vs. CT + TT), and heterozygote vs. homozygote (CT vs. CC + TT). We found no significant association between dominant (*P*=0.194) and recessive CT + TT (*P*=0.0442) genotypes or homo vs. het. (*P*=0.5) genotype models. The results of allele and genotype frequency and genotype association models are given in Table 1.

### 3.1. Effect of INSR rs1799817 SNP on Clinical, Hormonal, Biochemical, and Insulin Resistance Parameters

The effect of INSR C/T genetic variant on clinical, hormonal, metabolic, and biochemical parameters in PCOS cases and control women was analyzed by dominant (CC + CT vs. TT), recessive (CC vs. CT + TT), and additive (CT vs. CC vs. TT) genotype models. In genotype-phenotype association analysis using the dominant model, we found BMI was comparable (*P*=0.24) between CC + CT, 24.23 ± 4.74, and TT genotype 25.55 ± 4.37 in PCOS women. The level of cholesterol, triglycerides, testosterone, FG score, and FAI also had no significant variation (*P* > 0.05) in this genotype model. Although in variant homozygous genotype, TT, the concentration was higher, there was no significant difference when compared for glucose F (85.60 ± 8.51 and 88.35 ± 10.01, *P*=0.18), glucose 2nd hour (114.72 ± 18.08 and 122.18 ± 18.89, *P*=0.08), and insulin F (13.29 ± 6.88 vs. 15.29 ± 7.94; *P*=0.23). The insulin resistance marker, HOMA, was significantly higher in TT genotype (2.85 ± 1.62 vs. 3.71 ± 1.92, *P*=0.029). Similarly, QUICKI values were lower in PCOS women with TT genotypes (0.33 ± 0.02 vs. 0.32 ± 0.02, *P*=0.037), indicating lower insulin sensitivity in such women. In controls, although other metabolic, hormonal, and biochemical parameters were comparable in dominant genotype model, the cholesterol (133.58 ± 19.14 vs. 146.33 ± 17.29, *P*=0.03) and DHEAS (2.78 ± 1.29 vs. 3.83 ± 1.02; *P*=0.008) were significantly higher in TT genotype. The results are summarized in Table 2.

**Figure 1:**
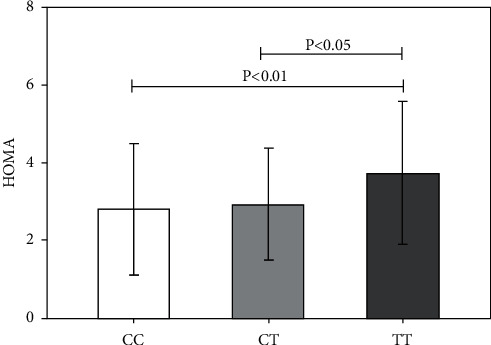
Comparison of the homeostatic model assessment of insulin resistance (HOMA-IR) values of INSR C/T SNP in CC vs. CT vs. TT genotypes. *P* values calculated by one-way ANOVA and intergroup differences tested by post hoc Tukey HSD test.

In recessive model, CC vs. CT + TT, no significant association was found in BMI (24.34 ± 4.72 vs. 24.17 ± 4.76, *P*=0.78), WHR (0.887 ± 0.08 vs. 0.88 ± 0.08, *P*=0.77), and testosterone (61.19 ± 20.59 vs. 61.59 ± 26.80, *P*=0.89). LH to FSH ratio was similar (*P*=0.85) in CC, 1.89 ± 1.08 compared to 1.92 ± 1.54, CT + TT genotype. The fasting insulin 13.07 ± 7.19 vs. 14.43 ± 6.57, *P*=0.13, fasting glucose 85.58 ± 8.48 vs. 86.12 ± 8.81, *P*=0.63, 2nd hour glucose 114.61 ± 18.21 vs. 116.19 ± 18.03, *P*=0.50, and HOMA 2.81 ± 1.70 vs. 3.10 ± 1.56, *P*=0.18, were also comparable. Systolic BP was significantly higher (*P*=0.039) in CT + TT genotype model with mean values of 119.39 ± 7.21 against 121.42 ± 7.60 for CC genotype. No significant change was observed in metabolic, clinical, or hormonal features of two genotypes in controls (Supplementary Table S1). In additive genotypic-phenotypic model, insulin resistance marker, HOMA, value was significantly higher in TT genotype carrier in PCOS women; CC vs. TT, *P* < 0.01, and CT vs. TT, *P* < 0.05 (Figure 1). Other biochemical, metabolic, and hormonal parameters were comparable across CC vs. CT vs. TT genotypes in PCOS women. In controls, DHEAs and cholesterol concentration was higher in TT genotypes (*P* < 0.05) but the rest of the tested parameters were comparable (Supplementary Table S2).

**Figure 2:**
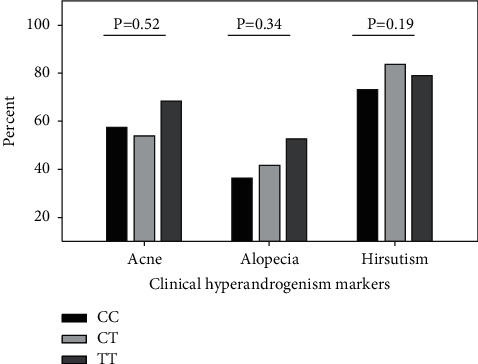
Comparison of INSR C/T SNP (rs1799817) genotypes with the presence of clinical manifestations of hyperandrogenism, acne, alopecia, and hirsutism in PCOS women. *P* value of CC vs. CT vs. TT calculated by Pearson chi-square test.

**Figure 3:**
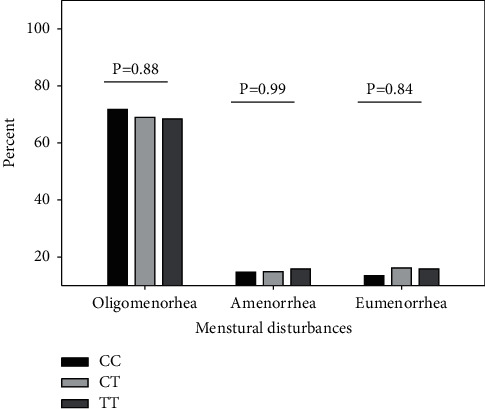
Comparison of the presence of menstrual disturbances, oligomenorrhea, and amenorrhea, in INSR C/T SNP genotypes in Kashmiri women with PCOS. *P* value of CC vs. CT vs. TT calculated by Pearson chi-square test.

**Figure 4:**
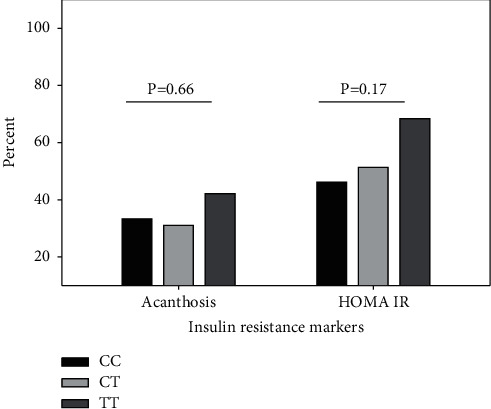
Prevalence of insulin resistance in women with PCOS as depicted by acanthosis nigricans and HOMA values >2.71 in CC, CT, and TT genotypes of INSR C/T SNP. *P* value of CC vs. CT vs. TT calculated by Pearson chi-square test.

In the analysis of INSR exon 17 C/T SNP (rs1799817) to study the effect of CC, CT, and TT genotypes on the clinical symptoms of PCOS, there was no significant association of this genetic variant with the tested parameters. The proportion of Kashmiri PCOS women with clinical markers of hyperandrogenism, acne, alopecia, and hirsutism was comparable across genotypes (Figure 2). No significant difference was found in the presence of menstrual dysfunction like oligomenorrhea (*P*=0.88) and amenorrhea (*P*=0.99) in such women (Figure 3). Although higher proportion of women with variant T allele had acanthosis nigricans (CC vs. CT vs.TT; 33.33% vs. 31.08% vs. 42.10%) (Figure 4) and body mass index greater than 25 kg/m^2^ (CC vs. CT vs.TT; 45.51% vs. 49.18% vs. 57.88%), the overall effect of this SNP on the metabolic parameters like obesity and insulin resistance was not statistically significant. The prevalence of obesity (BMI ≥30 kg/m^2^) in wild type vs. heterozygous vs. homozygous was 12.82% vs., 10.81% vs. 15.78%, respectively (Table 3).

## 4. Discussion

In the present study, INSR exon 17 C/T SNP (rs1799817, His1058) did not increase the risk of polycystic ovary syndrome in North Indian Kashmiri women. The tested SNP showed no significant association with different clinical symptoms of hyperandrogenism, menstrual, and metabolic dysfunction. The allele frequency was not significantly different between controls and PCOS cases (OR = 0.99, CI = 0.67–1.48, *χ*^2^ = 0.01, *P*=0.99). We found the frequency of the C allele was 77.51% in cases and controls, which is consistent with allele frequency reported in Caucasian PCOS women (71%) and similar to Azeri Iranian (63%) [25] and Croatian (80.33%) PCOS women [13]. The present study found no significant association (*χ*^2^ = 3.73, *P*=0.15) in genotypic distribution between PCOS and controls. The recessive, dominant, and homozygote vs. heterozygote association analysis also confirmed our results and showed no significant association in tested genotype models. Our results are consistent with other Asian studies: Indian (*P*=0.181) [26], Han Chinese (*P*=0.486), Iranian (*P*=0.964) [27], and Japanese (*P*=0.528) population [28]. No significant association was also found in Caucasian PCOS women: USA (*P*=0.32) [29], Croatian (*P*=0.96) [13], and English (*P*=0.96) [30] studies. A study on 186 PCOS cases and 156 controls of Iran also reported no association of INSR exon 17 C/T polymorphism with PCOS. However, contrary to our results, association of rs1799817 of INSR gene with PCOS was reported by GWAS studies conducted by Chen et al. and Shi et al. in Asian population [31, 32]. Gangopadhya et al. showed a significant association between PCOS and INSR rs1799817 SNP (*P* < 0.001) in small (cases/controls 50/50) Indian population [26]. A family association study investigating 260 family trios reported rs1799817 SNP is not significantly overtransmitted (*P*=0.48) to PCOS offspring from their parents [33]. The meta-analysis conducted by Feng et al. analyzed 20 INSR polymorphisms and PCOS case-control studies including 17,460 PCOS cases and 23,845 controls and concluded that 98 tested INSR SNPs showed no significant association with PCOS. In 12 case-control studies on rs1799817, the meta-analysis reported no significant association with PCOS (*P*=0.15) [20].  Table 4 summaries the results of previous association studies of rs1799817 SNP and compares them with the present study. Except for Mukherjee et al. [15] and Thangavelu et al. [22] who used SSCP sequencing and RT-PCR TaqMan assay, respectively, most of the previous studies employed PCR-RFLP analysis to study this genetic variant. Our study analyzed genotypic-phenotypic association of 249 PCOS women for this SNP which is the highest number of such women tested for this polymorphism (Table 4).

Further, the genotype-phenotype correlation analysis showed clinical, hormonal, and metabolic parameters were comparable in recessive and additive models. Mukherjee et al. also did not report any significant variation in phenotypic expression in CT + TT genotypes in comparison to CC genotypes in obese PCOS women [15]. We found the insulin resistance marker, HOMA, was significantly higher (*P*=0.029) in dominant genotype model CC + CT as compared to TT genotype. This is consistent with the increased level of insulin in CT + TT genotype and decreased insulin sensitivity. Though the increase in insulin level was not statistically significant (*P*=0.23), insulin sensitivity was significantly reduced (*P*=0.037) in variant TT genotype. Gangopadhya et al. reported increased serum insulin levels (*P* < 0.01) and HOMA (*P* < 0.05) in PCOS women with T allele [34]. A study including 180 PCOS women and 144 age-matched controls reported significant association of fasting insulin (*P*=0.02), HOMA (*P*=0.005), QUICKI (0.05), and FAI (*P*=0.004) in lean women with PCOS but these results were not replicated in obese PCOS women [15]. The prevalence of metabolic disturbances was higher in TT genotype but overall the effect on clinical symptoms of PCOS was not significant.

Our results suggest T allele may exert its effect on the phenotypic expression by its association with various other genetic variants and epigenetic modifications. This effect can be due to the increase in insulin-dependent serine phosphorylation of insulin receptor which can cause abnormal postreceptor activation and decrease response to insulin which in turn can lead to insulin resistance. Dunaif et al. reported ∼50% increase INSR phosphorylation in fibroblasts and skeletal muscles of women with PCOS [35]. Since the C/T His1058 SNP is a silent polymorphism, the effects on insulin sensitivity can be due to its linkage disequilibrium with other genetic variants. Urbanek et al. studied 367 families with at least one member with PCOS and reported significant association androgen level with chromosome location 19p13.3 and possible linkage with ELAV like RNA binding protein 1 (ELAVL1), Chemokine C-C motif ligand 25 (CCL25), and fibrillin 3 (FBN3) genes [36]. Although these genes are not apparently obvious candidate genes for PCOS, nearby regulatory elements, variable transcriptional activity, major splicing, and posttranslational modifications of these genes are controlled by dinucleotide repeat marker D19S884 which maps to ∼88 kb centromeric to INSR gene [37].

There are some limitations in our study. Although our study included the highest number of cases and controls compared to previous studies conducted on analyzing INSR gene exon 17 C/T SNP (rs1799817) in PCOS, we could not analyze the required number of controls to establish a significant powered study. Another limitation could be that the cases were recruited from a single tertiary healthcare institution which may not represent an unselected population and lead to a selection bias.

Although PCOS and its associated complications are complex polygenic and multifactorial conditions, it is generally considered as a disorder of hormonal dysfunction or fertility challenge. A recent meta-analysis of 101 randomized controlled trials investigated 55 drug interventions and reported comparable effect on various reproductive, metabolic, and hormonal parameters in obese/overweight PCOS women [38]. This shows that although there might be diverse clinical presentation of this syndrome, ultimately there must be major common pathway that amplifies the effects from various initial sources and leads to overall progression of PCOS. Hyperinsulinemia has not been as extensively considered as a primary trigger and unifying factor that can lead to development of PCOS. That is why even though insulin resistance remains ubiquitous with PCOS, it is still not used to guide the diagnosis of this syndrome. The impact of isolated genetic variants in such complex conditions is not easy to delineate. The genetic polymorphisms may only bring about modest changes in gene expression, regulation, and protein function. The effect of a single nucleotide polymorphism can become clinically relevant when it is simultaneously present with many genetic alterations and other predisposing environmental factors including life style and diet. Therefore, variants in insulin signaling genes and other genetic modulators of insulin signaling pathways may contribute to the development of a particular phenotypic manifestation of the syndrome.

## 5. Conclusion

We for the first time show that INSR exon 17 His1058 C/T (rs1799817) single nucleotide polymorphism is not associated with increased risk of PCOS in North Indian/Kashmiri women. This genetic variant does not play a significant role in increasing the cardiovascular and metabolic risk in PCOS. We found that HOMA value was significantly increased and QUICKI values were lowered in the presence of T allele. This suggests that although this SNP may increase the risk of insulin resistance in a subphenotype of PCOS in Kashmiri women, overall the tested genetic variant does not impart a significant effect on the clinical presentations of this syndrome.

## Figures and Tables

**Table 1:** Comparison of allele frequency, genotype distribution, and genotype association models of INSR exon 17 C/T SNP in PCOS and control women.

	Allele/genotype	Cases (*n* = 249)	Controls (*n* = 100)	Total (*n* = 349)	OR (95% CI)	*χ* ^2^ (*P*)
Allele frequency	C	386 (77.5%)	155 (77.5%)	541	1.00 (0.67–1.48)	0.01 (0.99)
	T	112 (22.5%)	45 (22.5%)	157		
Genotype distribution	CC	156 (62.65%)	67 (67.00%)	223 (63.90%)	—	3.73 (0.15)
	CT	74 (29.72%)	21 (21.00%)	95 (27.22%)		
	TT	19 (7.63%)	12 (12.00%)	31 (8.88%)		
Association models	CC + CT vs. TT	230 (92.4%) vs. 19 (7.6%)	88 (88%) vs. 12 (12%)	—	0.605 (0.28–1.29)	1.68 (0.19)
	CC vs. CT + TT	156 (62.7%) vs. 93 (37.3)	67 (67%) vs. 33 (33%)	—	1.21 (0.74–1.97)	0.58 (0.44)
	CT vs. CC + TT	33 (13.2%) vs. 216 (87.9%)	16 (16%) vs. 84 (84%)	—	1.25 (0.65–2.38)	0.45 (0.5)

PCOS: polycystic ovary syndrome, C T: alleles for INSR C/T polymorphism, CI: confidence interval, and Χ^2^: chi-square test with yates correction. Data of alleles are presented as number (%) of PCOS cases and controls. GG GA and AA are genotypes of IRS 1 (G/A) polymorphism in PCOS and controls, and Χ^2^: Pearson's chi-square test, (*P*) *P* value data of alleles is presented as number (%) of PCOS cases and controls.

**Table 2:** Comparison of anthropometric, clinical, hormonal, metabolic, and biochemical parameters in dominant genotype model for INSR rs1799817 SNP in PCOS women.

Parameter	PCOS		*P* value	Controls		*P* value
	CC + CT (*n* = 230)	TT (*n* = 19)		CC + CT (*n* = 88)	TT (*n* = 12)	
Age (years)	22.48 ± 4.19	21.88 ± 3.64	0.54	22.03 ± 3.15	21.83 ± 3.46	0.83
Weight (kg)	59.86 ± 11.71	61.53 ± 11.18	0.54	51.64 ± 6.61	54.17 ± 7.37	0.22
Height (m)	1.57 ± 0.05	1.55 ± 0.04	0.09	1.56 ± 0.05	1.58 ± 0.07	0.21
BMI (kg/m^2^)	24.23 ± 4.74	25.55 ± 4.37	0.24	21.08 ± 2.42	21.75 ± 2.89	0.38
Waist (cm)	83.17 ± 11.25	82.76 ± 10.74	0.87	76.59 ± 6.68	80.08 ± 9.47	0.11
Hip (cm)	93.39 ± 8.37	95.00 ± 7.57	0.48	90.82 ± 5.88	94.83 ± 8.46	0.03^*∗*^
WHR	0.89 ± 0.08	0.88 ± 0.07	0.59	0.84 ± 0.05	0.84 ± 0.06	1.00
WHtR	0.53 ± 0.07	0.53 ± 0.07	1.00	0.49 ± 0.04	0.51 ± 0.06	0.13
BAI	29.44 ± 4.78	31.18 ± 3.68	0.12	28.46 ± 3.01	29.80 ± 3.13	0.15
SBP (mmHg)	120.88 ± 7.45	118.41 ± 8.12	0.16	118.59 ± 5.49	121.67 ± 3.89	0.06
DBP (mmHg)	80.86 ± 5.87	80.65 ± 6.38	0.88	79.01 ± 5.34	81.58 ± 3.70	0.11
Menarche (yr)	13.14 ± 1.14	12.88 ± 1.17	0.34	13.30 ± 1.11	13.17 ± 0.83	0.69
FG score	13.99 ± 6.60	15.71 ± 6.59	0.27	4.59 ± 1.79	4.08 ± 2.23	0.37
LH (IU/L)	11.36 ± 9.82	10.15 ± 5.15	0.59	6.74 ± 2.37	6.00 ± 2.19	0.30
FSH (IU/L)	6.15 ± 1.85	5.77 ± 2.08	0.39	6.91 ± 1.99	6.83 ± 1.94	0.89
TT (ng/dL)	60.62 ± 21.28	72.31 ± 38.94	0.03	33.85 ± 15.06	36.32 ± 19.75	0.60
SHBG (nmol/L)	50.20 ± 21.75	49.31 ± 21.69	0.86	66.13 ± 25.51	55.97 ± 23.89	0.19
Andro (ng/mL)	3.26 ± 0.87	3.16 ± 0.79	0.62	2.22 ± 0.69	2.52 ± 0.62	0.15
DHEAS (ng/mL)	3.79 ± 1.16	3.74 ± 1.08	0.85	2.78 ± 1.29	3.83 ± 1.02	0.00^*∗*^
Insulin (*μ*IU/ml)	13.29 ± 6.88	15.29 ± 7.94	0.23	7.52 ± 4.78	9.17 ± 8.88	0.32
Glu F (mg/dL)	85.60 ± 8.51	88.35 ± 10.01	0.18	84.17 ± 8.97	87.25 ± 7.88	0.26
Glu 2 h (mg/dL)	114.72 ± 18.08	122.18 ± 18.89	0.08	108.23 ± 12.83	110.92 ± 23.00	0.54
Chol (mg/dL)	154.80 ± 35.04	156.00 ± 31.43	0.88	133.58 ± 19.14	146.33 ± 17.29	0.03^*∗*^
TG (mg/dL)	120.78 ± 35.62	119.88 ± 38.82	0.91	102.76 ± 14.54	102.50 ± 17.13	0.95
HOMA-IR	2.85 ± 1.62	3.71 ± 1.92	0.02^*∗*^	1.56 ± 1.01	2.04 ± 2.03	0.18
QUICKI	0.33 ± 0.02	0.32 ± 0.02	0.03^*∗*^	0.37 ± 0.04	0.39 ± 0.08	0.16
FAI	5.76 ± 5.72	6.33 ± 4.30	0.67	2.17 ± 1.70	2.53 ± 1.33	0.48
LH:FSH	1.88 ± 1.22	1.84 ± 1.09	0.89	1.05 ± 0.57	0.91 ± 0.29	0.40
LAP	35.51 ± 21.74	35.43 ± 22.71	0.98	21.69 ± 8.52	25.94 ± 12.29	0.12
Urea	22.63 ± 6.09	23.88 ± 5.19	0.38	21.34 ± 3.58	21.12 ± 2.62	0.83
Cre (mg/dL)	1.04 ± 0.43	0.85 ± 0.20	0.05	0.81 ± 0.14	0.78 ± 0.12	0.48
AST (U/L)	31.24 ± 12.40	36.12 ± 13.12	0.10	18.36 ± 8.20	17.85 ± 4.67	0.83
ALT (U/L)	28.23 ± 14.18	23.18 ± 7.11	0.12	23.22 ± 6.85	25.21 ± 6.29	0.34

Data presented as mean ± SD. ^*∗*^*P* value <0.05 significant. *P* values calculated by independent Student's *t* test. PCOS: polycystic ovary syndrome, BMI: body mass index, SBP: systolic blood pressure, DBP: diastolic blood pressure, men: menarche, FG score: Ferriman–Gallwey score, LH: luteinizing hormone, FSH: follicle-stimulating hormone, TT: total testosterone, PRL: prolactin, TSH: thyroid-stimulating hormone, SHBG: sex hormone-binding globin, andro: androstenedione, DHEAS: dehydroepiandrosterone sulfate, Glu F: glucose fasting, CHOL: cholesterol, TG: triglycerides, HOMA: IR homeostasis model assessment-estimated insulin resistance, QUICKI: quantitative insulin sensitivity check index, FAI: free androgen index, UA: uric acid, Cre: creatinine, AST: aspartate aminotransferase, and ALT: alanine aminotransferase.

**Table 3:** Association of INSR C/T SNP (rs1799817) genotypes with body mass index in PCOS.

Clinical parameter		CC (*n* = 156)	CT (*n* = 74)	TT (*n* = 19)	(*χ*^2^) *P*
Body mass index	Obese	20 (12.82%)	8 (10.81%)	3 (15.78%)	(0.39) 0.82
	Overweight	51 (32.69%)	21 (28.37%)	8 (42.10%)	(1.36) 0.50
	Normal	67 (42.94%)	33 (44.59%)	6 (31.57%)	(1.07) 0.58
	Underweight	18 (11.53%)	12 (16.21%)	2 (10.52%)	(0.78) 0.67

Data presented as number (percent). Clinical features defined as follows: obese BMI ≥30 kg/m^2^, overweight BMI ≥25–29.99 kg/m^2^, normal BMI ≥18.6–24.99 kg/m^2^, and underweight BMI ≤18.5 kg/m^2^, (*χ*^2^). *P*: *P* value of CC vs. CT vs. TT calculated by Pearson chi-square test.

**Table 4:** Comparison of the present study with previous studies of INSR gene exon 17 C/T (rs1799817) polymorphism and PCOS.

Author	Year	Ethnicity/country	Case/controls	*P* value
Conway et al. [39]	1994	Caucasian/British	22/8	1.00
Siegel et al. [21]	2002	Caucasian/USA	99/136	0.32 obese/0.03 lean
Talbot et al. [40]	2006	Caucasian/British	24/5	0.25
Lee et al. [41]	2006	Asian/South Korean	132/100	0.35
Unsal et al. [19]	2009	Caucasian/Turkish	44/50	0.53
Mukherjee et al. [15]	2009	South Asian/Indian	180/144	0.05
Ranjzad et al. [42]	2012	Asian/Iranian	181/181	0.42
Kashima et al. [28]	2013	Asian/Japanese	61/99	0.17
Tehrani et al. [27]	2013	Caucasian/Iranian	186/156	0.62
Skrgatić et al. [13]	2013	Caucasian/Croatia	150/170	0.86
Mutib et al. [43]	2014	Caucasian/Iraqi	84/65	<0.001^*∗*^
Bagheri et al. [44]	2015	Asian/Iranian	50/47	0.52
Feng et al. [20]	2015	Meta-analysis	1158/1264	0.48
Gangopadhyay et al. [26]	2016	South Asian/Indian	50/50	0.008^*∗*^
Shi et al. [32]	2016	Meta-analysis	1145/1065	0.66
Thangavelu et al. [22]	2017	South Asian/Indian	169/169	0.80
Present study	2021	South Asian/Kashmiri	249/100	0.15

## Data Availability

The datasets used and analysed during this study are available from the corresponding author on reasonable request.

## Supplementary Materials

Supplementary Materials Table S1: genotypic-phenotypic correlation of INSR C/T polymorphism in recessive model. Table S2: clinical characteristics, hormonal levels, and metabolic and biochemical profile of additive genotype model in PCOS women.
