# Supplementary material for: Clinical Manifestations of Hyperandrogenism and Ovulatory Dysfunction Are Not Associated with His1058 C/T SNP (rs1799817) Polymorphism of Insulin Receptor Gene Tyrosine Kinase Domain in Kashmiri Women with PCOS
Source: Int J Endocrinol. 2021 Dec 6;2021:7522487. doi: 10.1155/2021/7522487 (PMC8668320; doi:10.1155/2021/7522487)
Supplement: Supplementary Materials — Table S1: genotypic-phenotypic correlation of INSR C/T polymorphism in recessive model. Table S2: clinical characteristics, hormonal levels, and metabolic and biochemical profile of additive genotype model in PCOS women. [file 7522487.f1.docx]

**Supplementary material**

**Clinical manifestations of hyperandrogenism and ovulatory dysfunction are associated with His1058 C/T SNP (rs1799817) polymorphism of insulin receptor gene tyrosine kinase domain in Kashmiri women with PCOS**

Shayaq Ul Abeer Rasool^1^, Sairish Ashraf^2^, Mudasar Nabi^2^, Khalid M. Fazili^1^, Shajrul Amin^2*^

1. Department of Biotechnology, University of Kashmir, Srinagar India
2. Department of Biochemistry, University of Kashmir, Srinagar India

*Corresponding Author

**Shajrul Amin**

Professor & Head

Department of Biochemistry, University of Kashmir,

Srinagar-190006, India.

Tel: +91-9419018174

Email: shajrulamin@uok.edu.in

**Table S1: Genotypic-phenotypic correlation of INSR C/T polymorphism in recessive model**

| Parameter | PCOS | | P value | Controls | | P value |
| --- | --- | --- | --- | --- | --- | --- |
|  | CC (n=156) | CT+TT (n=93) |  | CC (n=67) | CT+TT (n=33) |  |
| Age (years) | 22.38 ±3.99 | 22.49 ±4.45 | 0.840 | 21.84± 3.00 | 22.36±3.51 | 0.443 |
| Weight (kg) | 60.16 ±11.55 | 59.38 ±11.93 | 0.611 | 51.30±6.66 | 53.24± 6.75 | 0.176 |
| Height (m) | 1.57 ±0.05 | 1.57 ±0.05 | 1.000 | 1.56±0.05 | 1.57±0.06 | 0.381 |
| BMI (kg/m^2^) | 24.34±4.72 | 24.17±4.76 | 0.784 | 21.02±2.56 | 21.46±2.30 | 0.406 |
| Waist (cm) | 83.15 ±11.29 | 82.97 ±10.83 | 0.902 | 75.91±6.45 | 79.24±7.92 | 0.027 |
| Hip (cm) | 93.59 ±8.37 | 93.22 ±8.19 | 0.734 | 90.51±5.72 | 92.91±7.26 | 0.074 |
| WHR | 0.887 ±0.082 | 0.89 ±0.08 | 0.778 | 0.84±0.05 | 0.85±0.06 | 0.381 |
| WHtR | 0.529±0.07 | 0.53±0.07 | 0.916 | 0.49±0.04 | 0.50±0.05 | 0.282 |
| BAI | 29.52±4.79 | 29.58±4.67 | 0.932 | 28.42±3.12 | 29.03±2.86 | 0.347 |
| Menarche (years) | 13.21 ±1.12 | 13.00 ±1.15 | 0.157 | 13.31±1.21 | 13.21±0.74 | 0.664 |
| FG Score | 13.62 ±6.73 | 14.80±6.34 | 0.172 | 4.52±1.88 | 4.55±1.79 | 0.939 |
| LH (IU/L) | 10.99 ±6.86 | 11.79 ±12.86 | 0.523 | 6.72±2.54 | 6.51±1.95 | 0.677 |
| FSH (IU/L) | 6.07 ±1.88 | 6.17 ±1.88 | 0.685 | 6.76±2.01 | 7.17±1.91 | 0.332 |
| TT (ng/dL) | 61.19±20.59 | 61.59 ±26.80 | 0.895 | 34.37±15.70 | 33.70±15.62 | 0.841 |
| TSH (uIU/L) | 3.29 ±1.51 | 3.01 ±1.29 | 0.138 | 2.97±1.17 | 3.02±1.96 | 0.873 |
| SHBG (nmol/L) | 51.12 ±22.88 | 48.39±19.35 | 0.336 | 67.74±24.28 | 59.16±27.08 | 0.113 |
| Andro (ng/mL) | 3.22 ±0.86 | 3.32 ±0.86 | 0.375 | 2.19±0.73 | 2.41±0.58 | 0.134 |
| DHEAS (ng/mL) | 3.82 ±1.11 | 3.74 ±1.24 | 0.599 | 2.72±1.32 | 2.28±1.20 | 0.109 |
| Insulin F (μIU/ml) | 13.07 ±7.19 | 14.43 ±6.57 | 0.137 | 7.93± 5.03 | 7.30±6.13 | 0.585 |
| Glu F (mg/dL) | 85.58 ±8.48 | 86.12 ±8.81 | 0.632 | 84.51±9.14 | 84.61±8.44 | 0.958 |
| Glu 2h (mg/dL) | 114.61±18.21 | 116.19 ±18.03 | 0.506 | 108.81±13.20 | 108.03±16.48 | 0.798 |
| Chol (mg/dL) | 154.96±36.79 | 155.09±30.90 | 0.977 | 134.97±19.19 | 135.39±19.83 | 0.919 |
| TG (mg/dL) | 120.81±37.31 | 120.77 ±32.82 | 0.993 | 102.97±14.98 | 102.24±14.58 | 0.817 |
| HOMA IR | 2.81 ±1.70 | 3.10±1.56 | 0.181 | 1.65±1.07 | 1.55±1.37 | 0.69 |
| QUICKI | 0.33 ±0.02 | 0.327 ±0.02 | 0.253 | 0.371±0.04 | 0.386±0.06 | 0.140 |
| FAI | 5.67 ±5.04 | 5.98 ±6.49 | 0.674 | 2.08±1.65 | 2.48±1.65 | 0.257 |
| LH:FSH | 1.89 ±1.08 | 1.92 ±1.54 | 0.857 | 1.09±0.64 | 0.93±0.21 | 0.165 |
| LAP | 35.21±22.66 | 34.66±20.35 | 0.847 | 20.97±8.33 | 24.70±10.12 | 0.053 |
| Urea | 22.75 ±5.91 | 22.72±6.24 | 0.969 | 21.45±3.70 | 21.05±2.98 | 0.590 |
| Creatinine (mg/dL) | 1.02 ±0.41 | 1.06 ±0.47 | 0.481 | 0.81±0.14 | 0.78±0.13 | 0.305 |
| UA (mg/dL) | 4.31 ±1.07 | 4.22± 1.13 | 0.53 | 3.72±0.67 | 4.08±0.85 | 0.023 |
| AST (U/L) | 32.12 ±12.19 | 30.47 ± 12.95 | 0.313 | 17.18±6.78 | 20.58±9.35 | 0.408 |
| ALT (U/L) | 27.27 ±13.34 | 28.79 ± 14.63 | 0.402 | 23.63±7.53 | 23.11±5.01 | 0.72 |
| Data presented as Mean ±SD. *P-value <0.05 significant. P values calculated by independent Student’s t test  PCOS polycystic ovary syndrome, BMI body mass index, SBP systolic blood pressure, DBP diastolic blood pressure, FG Score ferriman gallwey score, LH luteinizing hormone, FSH follicle stimulating hormone, TT total testosterone, PRL Prolactin, TSH thyroid stimulating hormone, SHBG sex hormone binding globin, Andro androstenrdione,DHEAS dihydroepiandrostenedione sulphate, Glu F glucose fasting, CHOL cholesterol, TG triglycerides, HOMA IR  homeostasis model assessment-estimated insulin resistance, QUICKI quantitative insulin sensitivity check index, FAI free androgen index, UA uric acid, AST aspartate aminotransferase, ALT alanine aminotransferase. | | | | | | |

Table S2: Clinical characteristics, hormonal levels, metabolic and biochemical profile of additive genotype model in PCOS women

| Parameter | PCOS | | | P value | Controls | | | P  value |
| --- | --- | --- | --- | --- | --- | --- | --- | --- |
|  | CC (n=156) | CT (n=74) | TT (n=19) |  | CC (n=67) | CT (n=21) | TT (n=12) |  |
| Age (yr) | 22.38 ±3.99 | 22.69 ±4.62 | 21.74 ±3.77 | 0.65 | 21.84± 3.00 | 22.66±3.58 | 21.83±3.46 | 0.56 |
| Weight (kg) | 60.16 ±11.55 | 59.21 ±12.10 | 60.00 ±11.53 | 0.84 | 51.30±6.66 | 52.71±6.50 | 54.17±7.37 | 0.33 |
| Height (m) | 1.57 ±0.05 | 1.57 ±0.05 | 1.55 ±0.04 | 0.33 | 1.56±0.05 | 1.57±0.05 | 1.58±0.07 | 0.51 |
| BMI (kg/m^2^) | 24.34±4.72 | 24.00 ±4.81 | 24.84 ±4.64 | 0.75 | 21.02±2.56 | 21.05±1.91 | 21.75±2.89 | 0.64 |
| Waist (cm) | 83.15±11.29 | 83.20±11.01 | 82.05±10.36 | 0.91 | 75.91±6.45 | 78.76±7.09 | 80.08±9.47 | 0.07 |
| Hip (cm) | 93.59 ±8.37 | 92.96 ±8.39 | 94.21 ±7.52 | 0.79 | 90.51±5.72 | 91.81±6.43 | 94.83±8.46 | 0.08 |
| WHR | 0.89 ±0.08 | 0.89 ±0.08 | 0.87 ±0.07 | 0.48 | 0.84±0.05 | 0.85±0.06 | 0.84±0.06 | 0.371 |
| WHtR | 0.53±0.07 | 0.53±0.07 | 0.53±0.07 | 0.99 | 0.49±0.04 | 0.50±0.04 | 0.51±0.06 | 0.15 |
| BAI | 29.52±4.79 | 29.30±4.84 | 30.63±3.84 | 0.54 | 28.42±3.12 | 28.42±2.76 | 29.80±3.13 | 0.34 |
| SBP (mmHg) | 121.42±7.60 | 119.73±7.03 | 118.05 ±7.9 | 0.08 | 118.60±5.99 | 118.57±3.58 | 121.67±3.89 | 0.18 |
| DBP (mmHg) | 81.24 ±5.82 | 80.05 ±5.93 | 80.05 ±6.49 | 0.30 | 78.85±5.75 | 79.52±3.84 | 81.58±3.70 | 0.26 |
| Men (yr) | 13.21 ±1.12 | 13.00 ±1.16 | 13.00 ±1.15 | 0.38 | 13.31±1.21 | 13.23±0.7 | 13.17±0.83 | 0.89 |
| FG Score | 13.62 ±6.73 | 14.77 ±6.30 | 14.89 ±6.70 | 0.39 | 4.52±1.88 | 4.81±1.47 | 4.08±2.23 | 0.55 |
| LH (IU/L) | 10.99 ±6.86 | 12.12±14.21 | 10.49 ±4.96 | 0.65 | 6.72±2.54 | 6.80±1.79 | 6.00±2.19 | 0.59 |
| FSH (IU/L) | 6.07 ±1.88 | 6.31 ±1.78 | 5.58 ±2.17 | 0.29 | 6.76±2.01 | 6.36±1.91 | 6.83±1.94 | 0.47 |
| TT (ng/dL) | 61.19±20.59 | 59.43±22.78 | 70.00±38.36 | 0.20 | 34.37±15.70 | 32.19±13.00 | 36.32±19.75 | 0.75 |
| TSH (uIU/L) | 3.29 ±1.51 | 2.99 ±1.32 | 3.06 ±1.22 | 0.30 | 2.97±1.17 | 3.21±2.38 | 2.69±0.84 | 0.60 |
| SHBG(nmol/L) | 51.12±22.88 | 48.27±19.15 | 48.88±20.61 | 0.62 | 67.74±24.28 | 60.97±29.14 | 55.97±23.89 | 0.24 |
| Andro (ng/mL) | 3.22 ±0.86 | 3.35 ±0.87 | 3.22 ±0.81 | 0.44 | 2.19±0.73 | 2.33±0.55 | 2.52±0.62 | 0.25 |
| DHEAS (ng/mL) | 3.82 ±1.11 | 3.72 ±1.28 | 3.81±1.06 | 0.84 | 2.72±1.32^b^ | 2.96±1.2 | 3.83±1.02 | 0.02* |
| Insulin(μIU/ml) | 13.07 ±7.19 | 13.75 ±6.18 | 17.05 ±7.51 | 0.06 | 7.93± 5.03 | 6.22±3.65 | 9.17±8.88 | 0.27 |
| Glu F (mg/dL) | 85.58 ±8.48 | 85.66 ±8.62 | 87.89±10.01 | 0.53 | 84.51±9.14 | 83.09±8.55 | 87.25±7.88 | 0.43 |
| Glu 2h (mg/dL) | 114.61±18.21 | 114.95±17.91 | 121.05±18.12 | 0.34 | 108.81±13.20 | 106.38±11.6 | 110.92±23.0 | 0.66 |
| Chol (mg/dL) | 154.96±36.79 | 154.47±31.29 | 157.47±30.03 | 0.94 | 134.97±19.19 | 129.14±18.7^c^ | 146.33±17.29 | 0.04* |
| TG (mg/dL) | 120.81±37.31 | 120.73±32.00 | 120.94±36.76 | 1.00 | 102.97±14.98 | 102.09±13.3 | 102.50±17.13 | 0.97 |
| HOMA IR | 2.81 ±1.70^b*^ | 2.94 ±1.44^c^ | 3.71±1.82 | 0.00* | 1.65±1.07 | 1.26±0.63 | 2.04±2.03 | 0.17 |
| QUICKI | 0.329 ±0.02 | 0.328 ±0.02 | 0.32 ±0.02 | 0.15 | 0.371±0.04 | 0.384±0.04 | 0.386±0.08 | 0.32 |
| FAI | 5.67 ±5.04 | 5.94 ±6.99 | 6.11 ±4.20 | 0.91 | 2.08±1.65 | 2.45±1.84 | 2.53±1.33 | 0.51 |
| LH:FSH | 1.89 ±1.08 | 1.86 ±1.48 | 2.17 ±1.76 | 0.60 | 1.09±0.64 | 0.93±0.18 | 0.91±0.29 | 0.38 |
| LAP | 35.43±22.71 | 35.03±20.55 | 33.21±20.01 | 0.91 | 20.97±8.33 | 23.99±8.91 | 25.94±12.29 | 0.13 |
| Urea (mg/dL) | 22.75 ±5.91 | 22.39 ±6.49 | 24.00 ±5.09 | 0.58 | 21.45±3.70 | 21.01±3.23 | 21.12±2.62 | 0.86 |
| Cre (mg/dL) | 1.02 ±0.41 | 1.09 ±0.49 | 0.95 ±0.36 | 0.34 | 0.81±0.14 | 0.78±0.14 | 0.78±0.12 | 0.62 |
| UA (mg/dL) | 4.31 ±1.07 | 4.14 ±1.15 | 4.54 ±1.05 | 0.29 | 3.72±0.67 | 4.07±0.91 | 4.11±0.75 | 0.07 |
| AST (U/L) | 32.12 ±12.19 | 29.39 ±12.73 | 34.68 ±13.27 | 0.15 | 17.18±6.78 | 22.14±10.99 | 17.85±4.67 | 0.03 |
| ALT (U/L) | 27.27 ±13.34 | 30.27 ±15.71 | 23.05 ±7.11 | 0.08 | 23.63±7.53 | 21.91±3.76 | 25.21±6.29 | 0.38 |
| Data presented as Mean ±SD. *P-value <0.05 significant. P values calculated by one way Analysis of Variance (ANOVA) independent standard weighted-means analysis and intergroup association tested by Post hoc Tukey HSD test.  PCOS polycystic ovary syndrome, BMI body mass index, SBP systolic blood pressure, DBP diastolic blood pressure, FG Score ferriman gallwey score, LH luteinizing hormone, FSH follicle stimulating hormone, TT total testosterone, PRL Prolactin, TSH thyroid stimulating hormone, SHBG sex hormone binding globin, Andro androstenrdione, DHEAS dihydroepiandrostenedione sulphate, Glu F glucose fasting, CHOL cholesterol, TG triglycerides, HOMA IR  homeostasis model assessment-estimated insulin resistance, QUICKI quantitative insulin sensitivity check index, FAI free androgen index, UA uric acid, AST aspartate aminotransferase, ALT alanine aminotransferase. | | | | | | | | |
